# Clinical effectiveness, implementation effectiveness and cost-effectiveness of a community singing intervention for postnatal depressive symptoms, SHAPER-PND: randomised controlled trial

**DOI:** 10.1192/bjp.2025.10377

**Published:** 2025-12

**Authors:** Rebecca H. Bind, Andrew J. Lawrence, Carolina Estevao, Katie Hazelgrove, Kristi Priestley, Lavinia Rebecchini, Riddhi Laijawala, Celeste Miller, Andy Healey, Joan Agwuna, Nick Sevdalis, Ioannis Bakolis, Rachel Davis, Maria Baldellou Lopez, Anthony J. Woods, Nikki Crane, Manonmani Manoharan, Alexandra Burton, Hannah Dye, Tim Osborn, Lorna Greenwood, Rosie Perkins, Paola Dazzan, Daisy Fancourt, Carmine M. Pariante

**Affiliations:** Clinical Postdoctoral Research Associate, Department of Psychological Medicine, Institute of Psychiatry, Psychology and Neuroscience, King’s College London, London, UK; Postdoctoral Image Analyst, Department of Psychosis Studies, Institute of Psychiatry, Psychology and Neuroscience, King’s College London, London, UK; Clinical Trial Manager, Department of Psychological Medicine, Institute of Psychiatry, Psychology and Neuroscience, King’s College London, London, UK; Postdoctoral Research Associate, Department of Psychological Medicine, Institute of Psychiatry, Psychology and Neuroscience, King’s College London, London, UK; PhD student, Department of Psychological Medicine, Institute of Psychiatry, Psychology and Neuroscience, King’s College London, London, UK; Research Assistant, Department of Psychological Medicine, Institute of Psychiatry, Psychology and Neuroscience, King’s College London, London, UK; Senior Research Worker, Centre for Implementation Science, Health Service and Population Research Department, Institute of Psychiatry, Psychology and Neuroscience, King’s College London, London, UK; Research Associate, Centre for Implementation Science, Health Service and Population Research Department, Institute of Psychiatry, Psychology and Neuroscience, King’s College London, London, UK; Professor, Centre for Implementation Science, Health Service and Population Research Department, Institute of Psychiatry, Psychology and Neuroscience, King’s College London, London, UK; Centre for Implementation Science, Health Service and Population Research Department, Institute of Psychiatry, Psychology and Neuroscience, King’s College London, London, UK; Programme Manager, Department of Psychological Medicine, Institute of Psychiatry, Psychology and Neuroscience, King’s College London, London, UK; Programme Lead, Culture Team, King’s College London, London, UK; Consultant Psychiatrist, Perinatal Psychiatry, South London and Maudsley NHS Foundation Trust, London, UK; Senior Research Fellow, Department of Behavioural Science and Health, University College London, London, UK; Head of Programmes, Breathe Arts Health Research, London, UK; Engagement and Support Officer, Breathe Arts Health Research, London, UK; Head of Scalability, Breathe Arts Health Research, London, UK; Professor, Centre for Performance Science, Royal College of Music, London, UK; Professor, Faculty of Medicine, Imperial College London, London, UK; Professor, Department of Psychosis Studies, Institute of Psychiatry, Psychology and Neuroscience, King’s College London, London, UK; Professor, Department of Behavioural Science and Health, University College London, London, UK; Professor, Department of Psychological Medicine, Institute of Psychiatry, Psychology and Neuroscience, King’s College London, London, UK

**Keywords:** Postnatal depression, group singing, arts health interventions, health economics, clinical effectiveness

## Abstract

**Background:**

Postnatal depression (PND) affects up to one in four mothers. However, they may experience barriers to access to conventional treatments, indicating a need for alternatives such as arts-based interventions. A previous trial showed that a 10-week singing intervention could alleviate symptoms of PND.

**Aims:**

To evaluate, in a larger sample and across a longer timeframe than previously, the clinical effectiveness, implementation effectiveness and cost-effectiveness of the Melodies for Mums (M4M) singing intervention for symptoms of PND.

**Method:**

One-hundred and ninety-nine mothers experiencing symptoms of PND (Edinburgh Postnatal Depression Scale score ≥10) and their babies were randomised to 10 weeks of in-person singing sessions (M4M, *n* = 133) or an active control (existing community-based mother–baby activities, *n* = 66). Mothers were re-assessed at weeks 6, 10, 20 and 36 for depression, healthcare use for themselves and their babies, and health-related quality of life according to the EQ5D-3. The perceived acceptability (Acceptability of Intervention Measure), appropriateness (Intervention Appropriateness Measure) and feasibility (Feasibility of Intervention Measure) of the activity were also assessed at week 6. Trial registration number: NCT04834622.

**Results:**

Mothers in both groups experienced attenuation of depressive symptoms by week 10; however, those in the singing group maintained lower EPDS scores than those in the control group at week 20 (10.7 *v*. 12.2 (mean difference 95% CI [−2.96, −0.22]), *P* = 0.023) and week 36 (9.85 *v*. 11.4 [−2.93, −0.19], *P* = 0.026). Mothers in the singing group were also more likely to remain in the study (77 *v*. 57%, *χ* ^2^(1) = 12.92, *P* < 0.001) and found their programme more acceptable (4.75 *v.* 4.0 [0.25, 0.83], *U* = 2436.5, *P* < 0.001), appropriate (4.25 *v*. 3.88 [0.12, 0.62], *U* = 2241.5, *P* < 0.001) and feasible (4.75 *v*. 4.0 [0.41, 0.91], *U* = 2568.0, *P* < 0.001). Finally, M4M was associated with 15 extra days of health and was found to be cost-effective (£126–539 per dyad).

**Conclusion:**

M4M had a long-lasting effect on symptoms of PND and was perceived to be more suitable than existing activities; thus, M4M represents a worthwhile investment for healthcare systems as an intervention for mothers experiencing symptoms of PND.

PND can affect up to 24% of mothers in the UK.^1^ Although standard treatments have lower-than-ideal uptake in this population, recent studies have highlighted the potential of community-based art interventions for PND.^2,3^ However, the real-life effectiveness of such approaches in the community has not been fully tested in a large sample in comparison with existing community interventions. Untreated PND has long-term effects not only on maternal well-being but also on infant developmental outcomes, as well as affecting the quality of the evolving mother–infant relationship.^4,5^ Given the surging rates of PND^6^ and its widespread implications for both mother and baby, it is of the utmost importance to identify and treat PND as early as possible. Although the gold standard for treatment is psychological therapy and psychopharmacology, many mothers with PND experience challenges and stigma in accessing care, at both individual and system levels.^7–10^

## Singing interventions for PND

Recent studies and a systematic review^11^ have highlighted the potential of community-based arts interventions for both psychological and physical health. More specifically, group singing has been found to alleviate symptoms of PND and anxiety, as mothers feel a sense of social support and interaction, stress reduction and relaxation.^12–14^ Furthermore, group singing has been found to promote bonding between a mother and their baby, as singing to one’s infant reduces psychological and physiological stress and fosters connection, an effect that has not been found in other psychosocial interventions for mothers with PND.^12,15^ Notably, given how effective group singing has been found to be for maternal mental health in the UK, the World Health Organization recently adapted and delivered a series of singing interventions for PND (Music and Motherhood) across multiple countries in Europe.^16^

Breathe Melodies for Mums (M4M) is a community singing intervention developed by Breathe Arts Health Research, a not-for-profit social enterprise, based on a three-arm randomised controlled trial (RCT) involving 134 mothers with symptoms of PND (with an Edinburgh Postnatal Depression Scale (EPDS) score ≥10 at baseline, indicating mild depression) and their babies.^14^ This initial trial compared the effectiveness of a 10-week group singing programme with an active control group (creative play classes for dyads) and a care-as-usual group. Mothers allocated to the singing intervention experienced significantly quicker improvement in their depressive symptoms than mothers receiving care as usual, an improvement not found in an active control group involving a bespoke non-musical play group.

## Present study

The Scaling-Up Health-Arts Programmes: Implementation and Effectiveness Research-Postnatal Depression (SHAPER-PND) study is a hybrid type 2 randomised clinical trial to assess on a large scale the clinical effectiveness, implementation effectiveness and cost-effectiveness of the M4M intervention (see protocol paper^17^). We had three objectives: first, to assess the clinical effectiveness of the M4M intervention in a larger sample and a longer timeframe than had been done previously, to understand whether it was more clinically effective than existing community-based activities, as well as whether it continued to be effective beyond the end of the 10-week intervention; second, to assess the acceptability, appropriateness and feasibility of M4M; and, third, to evaluate the cost-effectiveness of the intervention, including the cost of delivering the intervention, its impact on the cost of wider healthcare use by dyads and maternal quality-of-life outcomes.

Although the trial officially began in 2019, owing to the COVID-19 pandemic lockdowns and the urgent need for remote treatment options, we adapted the M4M intervention to an online platform, and, in a single-arm pilot (SHAPER-PNDO),^18^ assessed the feasibility and clinical effectiveness of a 6-week online singing intervention. Our results, albeit limited by the one-arm, uncontrolled design, showed quick and long-lasting improvements in mental health and well-being: mothers experienced significant decreases in symptoms of both depression and anxiety by week 3 of the intervention, effects that were sustained through the 8-month follow-up.^19^ Once lockdown restrictions had eased, we were able to move forward with the in-person SHAPER-PND trial. This paper reports on the primary objectives of the trial; secondary objectives, listed in our protocol, will be reported in subsequent papers.

## Method

### Study design

This was a multicentre trial that ran in children’s and community centres across south London (in the boroughs of Lewisham, Lambeth and Southwark). Participants were randomised at baseline in a 2:1 allocation of singing intervention to active control groups. For equity of access, mothers in the control group were offered singing following the 10-week intervention period, outside the study. All participants were followed up during the study period at 6 and 10 weeks and then again at 20 and 36 weeks post-baseline. The trial registration number is NCT04834622.

### Eligibility criteria

Mothers experiencing symptoms of PND, aged 18 or older, with a satisfactory understanding of English, with infants aged 0–9 months, with symptoms of depression (scoring ≥10 on the EPDS at screening, indicative of possible depression) were included.^14,20^ Participants required access to an internet-connected device (mobile phone, tablet, computer or laptop) to allow completion of assessments. Exclusion criteria were inability to give informed consent or already partaking in another singing-based activity.

### Protocol changes since registration

We originally included meeting the criteria for a clinical diagnosis of major depressive disorder in the postnatal period (according to the Structured Clinical Interview for DSM-IV (SCID-IV) criteria) as an inclusion criterion; however, this greatly limited our recruitment abilities and we subsequently removed it as an official protocol deviation.

### Study setting and recruitment

Mothers were recruited through social media advertising, posters and flyers in health visitor clinics and other community and clinical centres for postnatal mothers and their babies, signposting via health and social care professionals including general practitioners, midwives and mental health professionals in the South London and Maudsley NHS Foundation Trust, and the National Institute for Health Research (NIHR) Clinical Research Network. Recruitment took place in London, with a particular focus on the south London boroughs of Wandsworth, Lambeth, Southwark and Lewisham, as sessions were delivered in children’s centres and community venues in these areas.

**Fig. 1:**
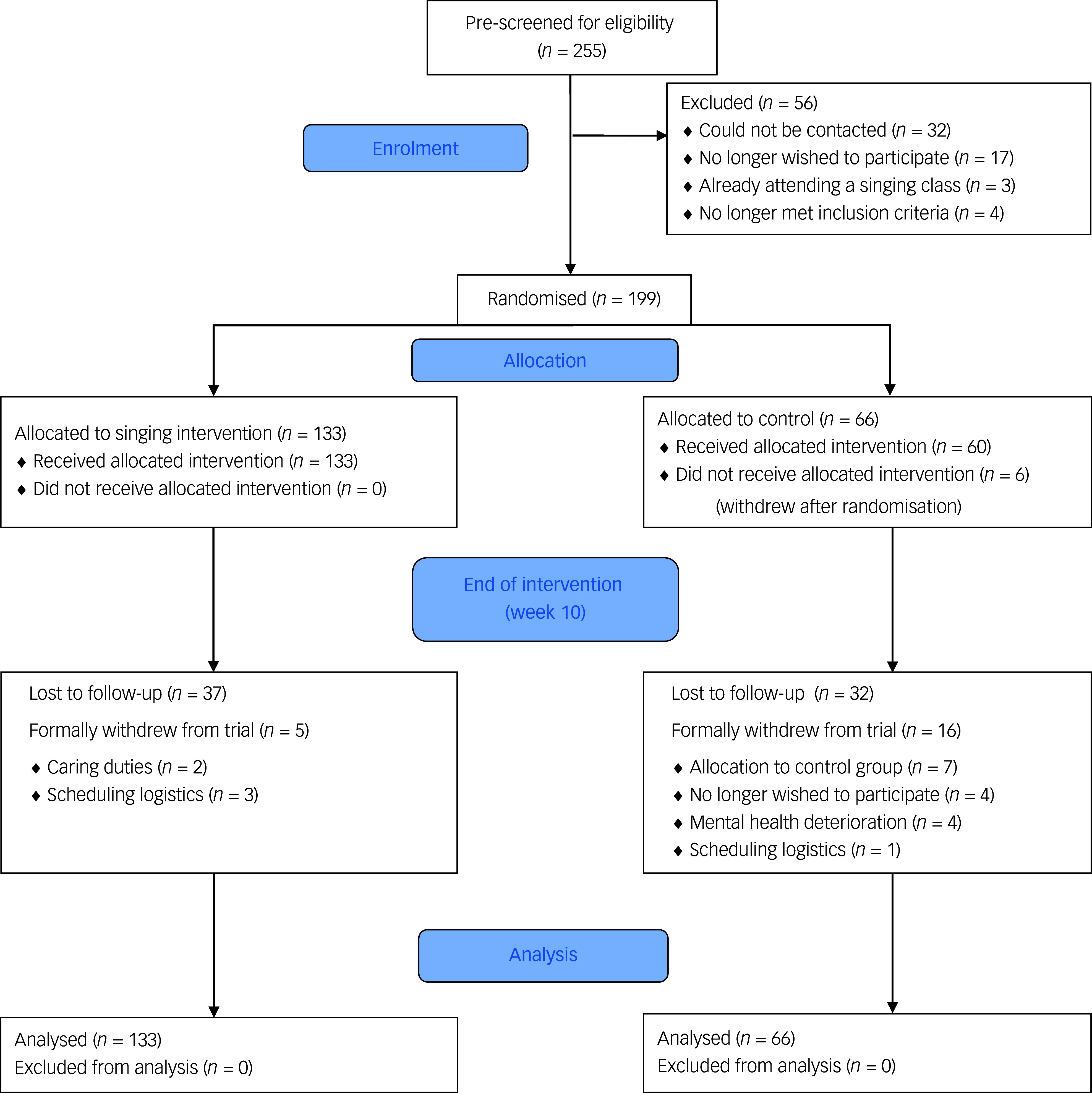
CONSORT 2010 flow diagram.

A total of 255 mothers met screening eligibility, and subsequently 199 mothers and their babies were determined to be fully eligible and enrolled in the trial across a total of seven cohorts of recruitment, from 20 September 2021 (recruitment of the first cohort) to 31 July 2024 (end of the study period for the last cohort). A CONSORT participant flow diagram across the study period is presented in Fig. 1. To enrol in the trial, mothers filled in an online form that included the EPDS. If their EPDS score was <10, they were notified that they were not eligible to participate and were signposted to other support services within the community (e.g. talking therapies, mother–baby groups, baby activity groups). They were also notified that they could rescreen for eligibility at the next round of recruitment. If eligible (EPDS ≥10), participants were sent the participant information sheet and consent form to review. Participants then provided consent and were enrolled in the trial before completing their baseline assessment and being randomised to either the singing intervention or the control group. Randomisation was performed using Sealed Envelope (version 1.23.1 on MacOS; Sealed Envelope, UK; https://www.sealedenvelope.com/),^21^ an online randomisation service, via a 2:1 intervention-to-control allocation process, following stratification of participants by EPDS eligibility score (10–13, 14–19, 20–30) and age of baby (0–3 months, 3–6 months, 6–9 months). It was important to balance the groups by EPDS scores as this has previously been found to influence the response to this intervention.^14^ The balance in the age of the baby was required for operational reasons, as groups needed a variety of ages to run smoothly. Given the nature of the intervention, both the mothers and research team were unblinded to group allocation. See the Supplementary Methods available at http://doi.org/10.1192/bjp.2025.10377 for sample size determination.

### Breathe M4M singing intervention group

Mothers and their babies who were allocated to M4M attended ten weekly, in-person, hour-long singing sessions, in groups of 8–12 dyads. Sessions were led by a specialist Breathe-trained creative health music lead and a Breathe staff member trained in safeguarding and working with vulnerable people. Mothers and their babies sat in a circle on the floor surrounded by soft play cushions and mats. Classes began with welcome songs and icebreakers, introducing mothers and their babies to one another, and then involved teaching a range of songs from across the world in different languages. Songs were sung in rounds with multiple parts and harmonies and accompanied by maracas, drums, and other simple instruments that mothers and their babies could play together. Mothers were reminded weekly by text or phone call of their session. Full details are available in a previous paper^17^ and the Supplementary Methods.

### Control group

Mothers in the ‘active’ control group were provided with details of other non-music mother–baby activities available in the community and received the same schedule of texts and phone calls as the intervention group to encourage them to join these activities. Following the 10-week study period, the mothers in the control group were then offered a place in the next singing cohort, outside the study. Mothers in the control group were regularly assessed by researchers and actively monitored for safeguarding concerns by researchers and Breathe, in the same way as the intervention group, throughout the trial.

### Clinical and social assessments

Study data were collected and managed using REDCap electronic data capture tools (version 15.0.36 on MacOS; Vanderbilt University, Nashville, Tennessee, USA; https://project-redcap.org/) hosted at King’s College London.^22^

### Demographics

At baseline, we collected demographic characteristics, as well as measures of risk factors for PND: the Threatening Life Experiences Questionnaire,^23^ the Child Experience of Care and Abuse Questionnaire,^24^ the Composite Abuse Scale-Pregnancy Version^25^ and the Intrusive Life Events Scale.^26^ A full description of the measures is available in the Supplementary Methods. The sociodemographic characteristics and risk factors are presented in Table 1. Despite randomisation, mothers in the singing group were less likely to be single (12 *v*. 26% *P* = 0.029) and to belong to an ethnic minority group (29 *v*. 47%, *P* = 0.027); however, these factors were not related to either drop-out rates or depressive symptoms throughout the study.

**Table 1:** Sociodemographic characteristics and risk factors of study participants

Characteristic or risk factor	Control group	Singing group	Statistic
Maternal age in years, mean (s.d.)	35.0 (4.9)	35.6 (4.4)	*P* = 0.43
Maternal marital status, *n* (%)			
Married or cohabiting	42 (73.7)	103 (88.0)	*P* = 0.029
Single with or without a partner	15 (26.3)	14 (12.0)	
Maternal ethnicity, *n* (%)			
Any White background	30 (52.6)	83 (70.9)	*P* = 0.027
Ethnic minority group	27 (47.4)	34 (29.1)	
Maternal qualifications, *n* (%)			
Higher education or above	48 (84.2)	103 (88.0)	*P* = 0.48
GCSE/A-level or below	9 (15.8)	14 (12.0)	
Maternal employment status, *n* (%)			
Employed, student or maternity leave	52 (91.2)	108 (92.3)	*P* = 0.78
Unemployed or full-time mother	5 (8.8)	9 (7.7)	
Household income, *n* (%)			
<£30 000	12 (23.1)	12 (10.6)	*P* = 0.06
≥£30 000	40 (76.9)	101 (89.4)	
Infant age in months, mean (s.d.)	4.0 (2.5)	3.6 (2.7)	*P* = 0.36
Infant gender, *n* (%)			
Female	28 (50.0)	61 (52.6)	*P* = 0.87
Male	28 (50.0)	55 (47.4)	
Attending other mother–baby group, *n* (%)			
Yes	36 (63.2)	79 (67.5)	*P* = 0.61
No	21 (36.8)	38 (32.5)	
Currently receiving any type of psychological therapy, *n* (%)			
Yes	14 (24.6)	40 (34.2)	*P* = 0.26
No	43 (75.4)	77 (65.8)	
Childhood experience of abuse, *n* (%)			
Yes	21 (36.8)	47 (40.5)	*P* = 0.74
No	36 (63.2)	69 (59.5)	
Adult experience of intimate partner violence, *n* (%)			
Yes	35 (61.4)	66 (57.4)	*P* = 0.36
No	22 (38.6)	49 (42.6)	
Intrusive life event across lifetime, *n* (%)			
Yes	31 (51.7)	72 (61.5)	*P* = 0.10
No	11 (18.3)	26 (22.2)	
Threatening life event in perinatal period, *n* (%)			
Yes	25 (41.7)	65 (55.6)	*P* = 0.06
No	17 (28.3)	34 (29.1)	
Lifetime diagnosis of major depressive disorder, *n* (%)			
Yes	41 (78.8)	86 (78.2)	*P* = 1.0
No	11 (21.2)	24 (21.8)	
Current diagnosis of major depressive disorder, *n* (%)			
Yes	21 (36.2)	32 (27.6)	*P* = 0.30
No	37 (63.8)	84 (72.4)	
Eligibility EPDS score, *n* (%)			
Below 13	15 (22.7)	19 (14.3)	*P* = 0.27
13 and above	51 (77.3)	114 (85.7)	

EPDS, Edinburgh Postnatal Depression Scale.

### Assessment of postnatal and lifetime depression

Our primary outcome measure was the EPDS,^20^ a validated questionnaire for PND, which was collected via online self-report questionnaires at eligibility screening and weeks 6, 10, 20 and 36 to assess the trajectory of symptoms of PND. To further evaluate present and past depression, the SCID-IV^27^ was administered to mothers at baseline to assess whether they met clinical criteria for major depressive disorder in the postnatal period, in their pregnancy, and at any other point in their lifetime. Several additional mental health and well-being measures were collected as secondary outcomes, together with biological samples and mother-infant videos; these are listed in the Supplementary Methods and in our protocol paper.^17^

### Implementation effectiveness

Quantitative data on the implementation effectiveness were collected using: the perceived Acceptability of Intervention Measure, the perceived Intervention Appropriateness Measure and the perceived Feasibility of Intervention Measure.^28^ More details are provided in the Supplementary Methods. The qualitative interviews that were conducted have been reported in a separate paper.^29^

### Cost-effectiveness

Our economic evaluation (a cost-utility analysis) was from the perspective of the programme payer, which was assumed to be the National Health Service (NHS) or local authority public health department. It drew on maternal-reported self and infant healthcare use and reported maternal health-related quality of life data (EQ5D-3L).^30^ A published tariff of ‘utility’ weights was used to convert the EQ5D data into quality-adjusted life years (QALYs) over 36 weeks (area under the curve method).^31^ National unit costs were used to value reported healthcare contacts (general practitioners and hospital-based contacts and maternal use of NHS psychological treatment), and M4M intervention costs were supplied by Breathe.^32,33^ All costs were estimates and reported at 2023–2024 price levels. Economic outcomes were undiscounted (time horizon less than 1 year). A detailed description of the economic data and statistical methods employed in the economic evaluation are included in the Supplementary Methods.

### Statistical analyses

Descriptive analyses were conducted with SPSS Statistics (version 27 for MacOS; IBM, UK; https://www.ibm.com/products/spss-statistics), and linear mixed-effects models with multiple imputation were conducted with R (version 4.4.2 for MacOS; R Core Team; https://www.r-project.org/) using the lme4 (v1.1.35.5), and emmeans (v1.10.6) packages. Before analysis, data were checked for normality. Demographics were analysed using frequencies and descriptives. A more detailed description of the statistical analyses, including imputation procedures, is presented in the Supplementary Methods.

As described in the protocol, our primary objective was to test whether singing reduced the severity of symptoms of PND according to the EPDS between baseline and week 10 (end of intervention) by paired tests, followed by a comparison of this outcome between the two groups, with an intention-to-treat (ITT) approach. We estimated the treatment effect of the singing group relative to the control group on the changes in EPDS score from eligibility assessment to weeks 6, 10, 20 and 36, adjusting for important determinants of recovery at baseline identified *a priori*: (a) history of childhood abuse (Child Experience of Care and Abuse Questionnaire); and (b) diagnosis of major depressive disorder (antenatal and postnatal). We included all post-randomisation EPDS scores as the model outcome and tested treatment effects on change through inclusion of eligibility EPDS as a linear covariate. Missing outcome data were dealt with using two approaches: (a) as per protocol, with ITT using the last observation carried forward approach; and (b) with all available data. Implementation effectiveness measures were summed and averaged for each measure, with medians and min–max ranges reported. As data were non-parametric, group medians were compared using Mann–Whitney *U*-test.

### Ethics statement

Ethical approval was granted by the London-West London and GTAC Research Ethics Committee (ref: 20/PR/0813), IRAS project ID 278445. The authors assert that all procedures contributing to this work comply with the ethical standards of the relevant national and institutional committees on human experimentation and with the Helsinki Declaration of 1975, as revised in 2008. Written informed consent was obtained by researchers from all participants involved in the study. All data were handled in line with the General Data Protection Regulation and other relevant regulations. Furthermore, all data were securely stored either in lockable cabinets or on encrypted hard drives or servers approved by the research institution and ethics committee. Only the research team members had access to the data.

## Results

### Participant retention was higher in the singing group

At baseline, 133 mothers were allocated to the singing intervention and 66 were allocated to the control group. Throughout the study, the retention rate was higher in the singing group than in the control group: at week 6 (89 *v*. 62%, *χ* ^2^(1) = 29.01, *P* < 0.001), week 10 (end of intervention; 77 *v*. 57%, *χ* ^2^(1) = 12.92, *P* < 0.001), week 20 (66 *v*. 55%, *χ* ^2^(1) = 4.38, *P* = 0.04) and week 36 (66 *v*. 38%, *χ* ^2^(1) = 17.54, *P* < 0.001). In addition, fewer mothers in the singing group formally withdrew from the study (4 *v*. 24% *χ* ^2^(1) = 19.61, *P* < 0.001). Of note, there was no effect of eligibility EPDS score (*t*(197) = 0.66, *P* = 0.51) or clinical (SCID-diagnosed) antenatal depression or PND (*χ* ^2^(1) = 0.71, *P* = 0.69) on retention; mothers in both groups who remained in the study were more likely to have experienced childhood maltreatment (*χ* ^2^(1) = 7.07, *P* = 0.006), perhaps suggesting that they felt supported by being in the study.

### PND symptoms improved with the singing intervention, but symptoms were only lower than that of controls at the 20- and 36-week follow-ups

The primary per-protocol analysis found that singing did reduce EPDS scores between baseline (16.7 ± 3.8) and week 10 (end of intervention) using both the last observation carried forward approach (11.3 ± 5.0; *t*(132) = 12.76, *P* < 0.001, 95% CI of the difference [0.89, 1.32]) and all available data (10.1 ± 4.7; *t*(101) = 13.12, *P* < 0.001, 95% CI [1.03, 1.56]). We subsequently compared this outcome between the two groups.

**Fig. 2:**
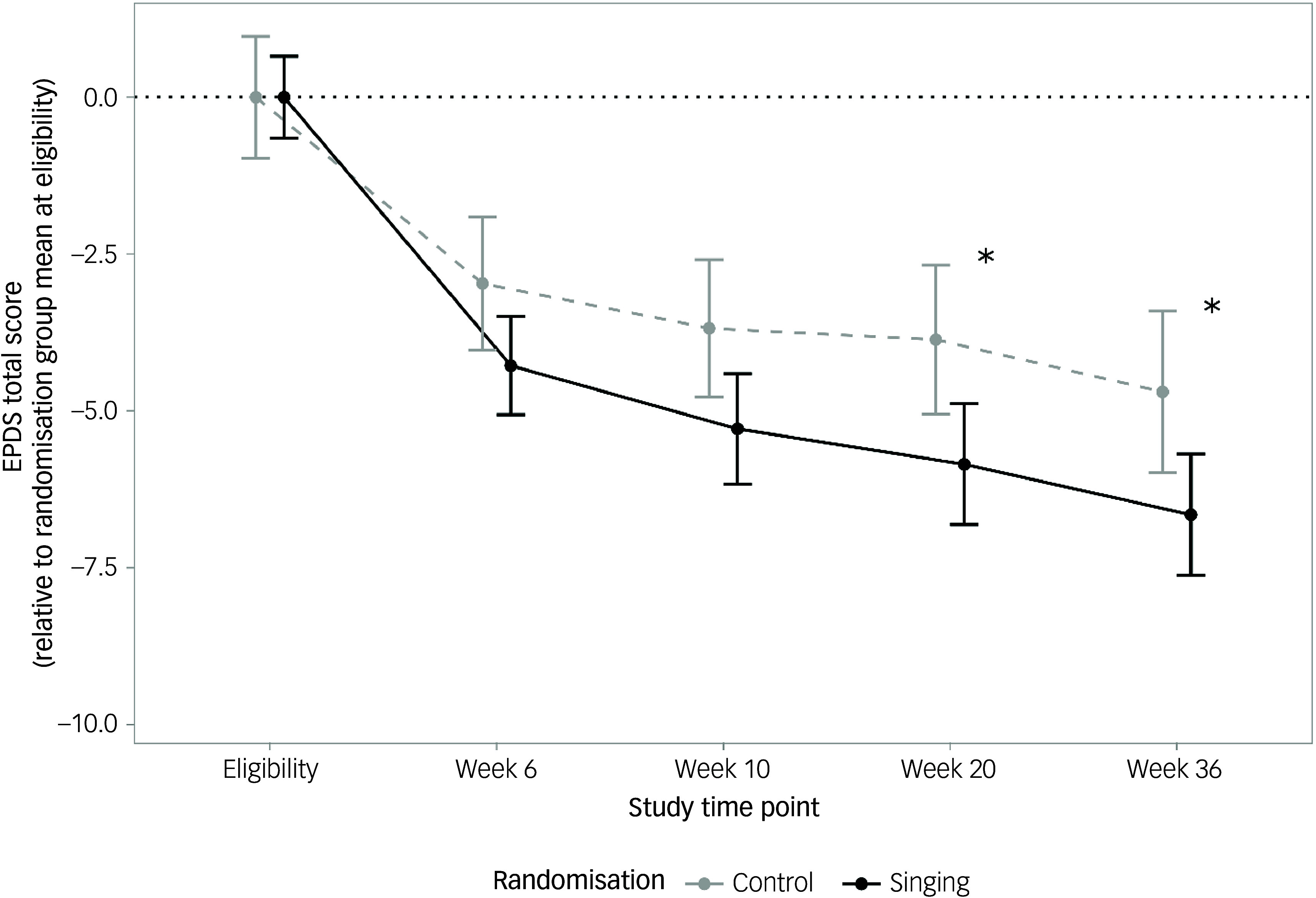
Mean changes in Edinburgh Postnatal Depression Scale (EPDS) score at weeks 6, 10, 20 and 36 relative to randomisation group mean at eligibility using last observation carried forward approach. *P < 0.05.

Results from the ITT approach are presented in Fig. 2, which shows EPDS scores relative to each group’s mean score at eligibility. There was no difference in EPDS score at eligibility between mothers in the singing and control groups (16.8 ± 3.8 *v*. 15.9 ± 4.0, *T*(197) = −1.54, *P* = 0.124). We found a statistically significant overall treatment effect of the singing intervention relative to the control activity (singing: weeks 6–36 mean EPDS: 12.3; control: weeks 6–36 mean EPDS: 11.0; treatment effect of singing: −1.31, s.e. = 0.63, 95% CI [−0.076,−2.55]; *F*(1,783) = 4.52, *P* = 0.034).

To understand at which time point(s) the group difference in EPDS occurred, we ran a *post hoc* analysis and found that EPDS scores did not significantly differ between groups at week 6 (i.e. mid-intervention: singing EPDS: 12.2 ± 0.4; control EPDS: 13.1 ± 0.6; treatment effect of singing: −0.91, 95% CI [−2.28, 0.46], *P* = 0.193) or at week 10 (end of intervention, EPDS: 11.2 ± 0.4 *v*. 12.4 ± 0.6; treatment effect: −1.20 95% CI [−2.57, 0.17], *P* = 0.085); however, mothers in the singing group had significantly lower EPDS scores at both week 20 (EPDS: 10.7 ± 0.4 *v*. 12.2 ± 0.6; treatment effect: −1.59, 95% CI [−2.96, −0.22], *P* = 0.023) and week 36 (EPDS: 9.85 ± 0.4 *v*. 11.4 ± 0.6; treatment effect: −1.56 95% CI [−2.93, −0.19], *P* = 0.026), indicating a sustained effect of the singing intervention. We did not find a significant omnibus effect for the interaction between time and group allocation (*F*(1,780) = 0.87, *P* = 0.46).

**Fig. 3:**
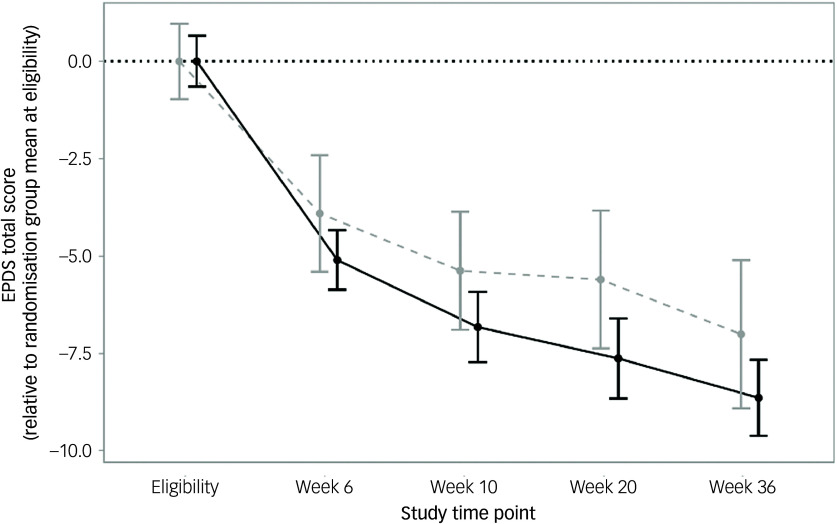
Mean changes in Edinburgh Postnatal Depression Scale (EPDS) score at weeks 6, 10, 20 and 36 relative to randomisation group mean at eligibility using available data.

Results from the available data (*n* = 146) are presented in Fig. 3. The main effect was in the same direction as in the ITT model, that is, lower EPDS scores in the singing group; however, this was not statistically significant (singing, weeks 6–36 mean EPDS 9.8, control, weeks 6–36 mean EPDS 10.7; treatment effect of singing: −0.94, s.e. = 0.72, 95% CI [−2.37, 0.485]; *χ* ^2^(1) = 1.56, *P* = 0.21). As before, the time × group interaction was non-significant (*χ* ^2^(3) = 2.28, *P* = 0.52).

### Breathe M4M was a more suitable intervention than the control activities

Compared with the community-based control activity, mothers in the singing group found the M4M programme to be significantly more (and very highly) acceptable (Acceptability of Intervention Measure: 4.75 *v*. 4.0 95% CI [0.25, 0.83], *U* = 2436.5, *P* < 0.001), appropriate (Intervention Appropriateness Measure: 4.25 *v*. 3.88 95% CI [0.12, 0.62], *U* = 2241.5, *P* < 0.001) and feasible (Feasibility of Intervention Measure: 4.75 *v*. 4.0 95% CI [0.41, 0.91], *U* = 2568.0, *P* < 0.001).

### Breathe M4M was a cost-effective intervention

On average, mothers in the singing group accumulated 0.041 additional QALYs over the 36-week study period (with adjustment for baseline scores) compared with those in the control group (95% CI [0.009, 0.072]): this indicates that mothers in the singing group gained around 15 additional days in full health over the 36-week study period. Detailed tabulated results are reported in the Supplementary Methods and Supplementary Tables, including levels of missing economic outcome data in the trial and sensitivity analyses.

Estimated costs of the intervention varied between £126 (lower value) and £539 (upper value) per mother–baby pair recruited. The upper value included the costs of all activities and expenditure arising from programme implementation and delivery; the lower value was an alternative estimate based only on the cost of direct delivery of singing sessions and provider overheads. The alternative value was intended to facilitate like-for-like comparisons with published trial-based incremental cost-effectiveness ratios for other programmes. In addition, the average total cost of reported care contacts was higher in the intervention group (baseline-adjusted difference of £331), but this was estimated with a wide margin of uncertainty (95% CI [−£220, £926]).

The total incremental cost (NHS intervention plus wider care contact costs) per maternal QALY gained ranged from £11 122 to £21 215, depending on whether the lower or the upper intervention payer cost was applied. This range almost entirely fell below what the UK healthcare regulator (the National Institute for Health and Care Excellence) recommends the NHS should pay for healthcare programmes (a maximum of £20 000 to £30 000 per QALY gain^34^).

## Discussion

SHAPER-PND is the first trial, to our knowledge, to evaluate the clinical effectiveness, implementation effectiveness and cost-effectiveness of group singing sessions for mothers experiencing symptoms of PND. We overall demonstrate that the M4M singing programme is clinically effective at reducing symptoms of PND across a 36-week period, that mothers find it to be a more suitable intervention than existing community-based mother–baby activities, and that it is a good investment for healthcare systems and local authorities from a cost-effectiveness perspective.

A noteworthy finding from our trial was that mothers who participated in the M4M intervention were more likely to attend their sessions, consistent with the results of a recent study which found mothers with PND who were offered a group singing intervention attended on average 90% of sessions.^16^ Together with our implementation results, which showed that mothers found the M4M intervention to be significantly more acceptable, appropriate and feasible than the control activity, these findings indicate that M4M is more engaging and accessible to mothers with PND than community mother–baby activities. These findings have been replicated by a study led by the World Health Organization that reported very high rates of acceptability, appropriateness and feasibility of group singing sessions for mothers with PND.^16^ Given that mothers’ severity of PND symptoms and prior experience of childhood maltreatment – two major predictors of overall functioning in the postnatal period^35,36^ – did not contribute to the differential withdrawal from the study, it is likely that disengagement was due to dissatisfaction with allocation to the control group. This would be consistent with the results of previous studies showing that attendance of community-based mother–infant and social groups is typically low for mothers experiencing PND.^37,38^

We successfully met our per-protocol primary clinical outcome, as we found that the M4M intervention effectively reduced symptoms of PND on the EPDS at week 10; furthermore, mothers in the singing group had significantly lower EPDS scores at weeks 20 and 36. These results confirm and expand the findings of the previous, smaller RCT,^13^ which found that mothers who participated in the singing group experienced a faster reduction in depressive symptoms compared with a care-as-usual group during the 10-week intervention period, a difference not found in the active control group. Notably, this previous study also found no significant differences between singing and the active control within the 10 weeks of the intervention, although our results here suggest that differences may occur after the interventions (however, it is important to note the active control in the previous study involved a bespoke 10-week non-music play group, whereas the present study signposted participants to a variety of different non-music play groups in the community). More recently, a systematic review also found that mothers with PND who partook in group singing experienced a greater decline in depressive symptoms than those in a control group.^11^ We can now identify persistent, protective effects of the singing intervention compared with the active control group, lasting for up to 6 months after the end of the intervention. Of course, the improvement in the control group during the intervention was not unexpected, as previous studies have found that attending mother–baby groups, even if not specifically for PND, can still provide mothers with social support,^39^ a great predictor of improvement.^40^ However, after week 10, and in the absence of this social support, the active control group activity was unable to deliver the same sustained effect on mothers’ mental health. There are thus additional mechanisms present in M4M singing sessions that the control activities cannot provide.^41^

First, there are therapeutic mechanisms present within the singing activity itself. For example, it is possible that the skills mothers learn during the singing sessions help them to care for their baby, thereby reducing depression. Indeed, in the previous RCT, it was found that mothers employed the songs learned in the classes at home as a way to soothe their babies, stop them crying and help them get to sleep, contributing to a greater sense of overall competency.^41^ Studies have also shown that the act of mothers singing to their babies reduces cortisol levels^12^; given that perinatal depression is associated with heightened cortisol output,^42^ it is possible that the reduction in cortisol through singing attenuates depressive symptoms. Furthermore, studies have found that group singing leads to increased oxytocin output,^43^ and depressive symptoms are associated with both reduced oxytocin^44^ and an impaired mother–infant relationship. Thus, it is possible that singing enhances mother–infant bonding in mothers with PND and thus ameliorates depression. Finally, it is also noteworthy that the singing sessions (but not the active control activities) were tailored specifically to mothers experiencing PND; a previous study^39^ found that PND-specific groups allowed mothers to connect with and receive support from other mothers going through experiences similar to their own. This notion is further supported by qualitative findings from semi-structured interviews in the present sample, in which mothers shared that they greatly valued the opportunity to build social connections in a group setting with other mothers going through similar mental health difficulties.^29^

Our findings offer encouraging evidence that referral to community singing groups represents a cost-effective addition to the care pathway for mothers experiencing symptoms of PND. The evidence becomes more uncertain at higher assumed costs to the NHS or a local authority payer; however, our ‘best’ central estimates suggested that the added cost per QALY gained would broadly fall below the maximum acceptable range applied by the National Institute for Health and Care Excellence. Additional sensitivity analysis (see the Supplementary Tables) revealed that value for money could be compromised with lower levels of population reach, particularly where the NHS or local authority pays most or all costs borne by the provider; thus, monitoring of recruitment levels should be an important component of delivery, to maintain value. The average total cost of reported care contacts was higher in the intervention group, but this is not necessarily negative, as greater engagement with primary care services may reduce demand for and costs of secondary and tertiary care. When comparing the range of incremental cost per QALY values estimated here (£11 122 to £21 215) with published values, including those reported in a recent systematic review,^45^ the costs of M4M were moderately higher than those reported for telephone-based peer support (£6768)^46^, comparable with those of structured psychological therapy (£17 481)^47^ and a psychoeducational programme (£21 987)^48^, and considerably lower than those of group cognitive–behavioural therapy (£39 875)^49^ and listening home visits (£66 275).^47^

The two major strengths of our study were: (a) the three-pronged approach to assessing the clinical effectiveness, implementation effectiveness and cost-effectiveness of the singing intervention; and (b) the 36-week timeframe of the study, which enabled us to track symptom progression far beyond the 10-week intervention period. In terms of limitations, we recognise that the results from the EPDS must be taken with the caveat that our strongest findings were obtained using a (per-protocol) ITT approach. However, given that we have strong evidence to suggest that attrition was due to group allocation, and considering that the effect in the available-data analysis was in the same direction, we believe ours to be a valid approach. Of course, we had greater retention in the intervention group than in the control group, possibly introducing bias; however, our use of the ITT approach was also intended to mitigate any sample bias by preserving all participants’ data. Furthermore, we recognise that we modified our inclusion criteria so that participants did not need to meet clinical criteria for a major depressive episode; as such, we evaluated the efficacy of our intervention for a wide range of PND symptoms, limiting the generalisability of our findings to populations with clinically diagnosed PND. Future studies could investigate whether this intervention is similarly effective in a sample of mothers with clinically diagnosed depression. In addition, our recruitment fell short of our initial target (Supplementary Methods), and we also experienced attrition of 69 participants by the end of the study period, ultimately underpowering our study. Nevertheless, much of our recruitment difficulty stemmed from COVID-19, as the usual in-person recruitment pathways that reach the most mothers, such as health visiting clinics or community venues, were restricted. Importantly, our sample was still significantly more powered than the previous RCT (199 participants across two arms here versus 134 across three arms previously).^14^ Specific limitations of the economic evaluation are presented in the Supplementary Discussion.

Overall, the SHAPER-PND trial provides evidence of clinical effectiveness, implementation effectiveness and cost-effectiveness of the Breathe M4M singing intervention for mothers experiencing symptoms of PND. Given this evidence, in addition to the findings that the effects of M4M on symptoms of PND are longer lasting and that mothers are more likely to attend these sessions than other community activities, we believe there would be great value in health and social care systems investing in this intervention.

## Supporting information

Bind et al. supplementary material 1 Bind et al. supplementary material

Bind et al. supplementary material 2 Bind et al. supplementary material

## Data Availability

The data and research material that support the findings of this study are available from the King’s Open Research Data System upon reasonable request (https://doi.org/10.18742/27256014.v1). The analytic code used for data analysis is available from the corresponding author, R.H.B., upon reasonable request.

## Supplementary material

The supplementary material is available online at https://doi.org/10.1192/bjp.2025.10377
