# Supplementary material for: Clinical effectiveness, implementation effectiveness and cost-effectiveness of a community singing intervention for postnatal depressive symptoms, SHAPER-PND: randomised controlled trial
Source: Br J Psychiatry. 2025 Oct 15;227(6):836–45. doi: 10.1192/bjp.2025.10377 (PMC12628126; doi:10.1192/bjp.2025.10377)
Supplement: Bind et al. supplementary material 1 — Bind et al. supplementary material [file S0007125025103772sup001.docx]

**Supplementary Tables**

**Table S1: Provider cost of implementing and delivering 20 singing sessions over 10 weeks**

| **Resource item** | **Cost** | **Notes/assumptions** | **Table row number** |
| --- | --- | --- | --- |
| **Total cost of local area scoping activity** | **£3,626** |  | 1 |
| **Total indirect cost** | **£9,921** | Includes staffing costs attached to programme development and recruitment, marketing costs, safeguarding, participant stewardship, artist training/planning and a contribution to organisational overheads. | 2 |
| **Total direct delivery cost** | **£2,630** | Includes staffing costs attached to session delivery (Breathe staff member and artist), participant access support costs, refreshments and equipment. Venue is offered in kind by Children and Family Centres. | 3 |
| **Total programme cost** | **£16,177** | = row 1+row 2 + row 3 | 4 |
| **Provider programme cost per mother/baby dyad: Upper** | **£540** | = row 4 ÷ 30 mother/baby dyads | 5 |
| **Provider programme cost per mother/baby dyad: Lower** | **£126** | = (% of row 2 reflecting contribution to organisational overheads + row 3) ÷ 30 mother/baby dyads | 6 |

**Table S2: Unit costs of health care contacts**

|  | **Unit cost** |
| --- | --- |
| GP visit | £38.45 per visit |
| A and E attendance | £180.08 per attendance |
| Outpatient attendance | £250.99 |
| Inpatient admittance (maternal) | £1018.63 per admission |
| Inpatient admittance (infant) | £930.89 per admission |
| NHS psychological therapy attendance | £118.57 per attendance |

**Table S3: number of randomised cases (%) included in analysis (N=177)^1^ with missing cost and outcome data**

|  | Baseline | | 6 weeks | | 10 weeks | | 20 weeks | | 36 weeks | |
| --- | --- | --- | --- | --- | --- | --- | --- | --- | --- | --- |
|  | Singing | Control | Singing | Control | Singing | Control | Singing | Control | Singing | Control |
| Total cost | 1 (<1%) | 5 (8%) | 14 (12%) | 26 (43%) | 31 (26%) | 30 (50%) | 40 (34%) | 35 (58%) | 48 (41%) | 43 (72%) |
| EQ5D-3L utilities | 23 (20%) | 20 (33%) | 11 (9%) | 26 (43%) | 117 (100%) | 60 (100%) | 40 (34%) | 36 (60%) | 46 (39%) | 42 (70%) |

1. 117 randomised to singing sessions and 60 randomised to the control group

**Table S4: Weekly cost of health care contacts (mother and baby) and maternal E5D-3L utility scores (descriptive statistics)**

|  | Maternal health care contacts: cost per week | Maternal NHS psychological therapy contacts: cost per week | Infant health care contacts: cost per week | Maternal EQ5D-3L utility score at end of period |
| --- | --- | --- | --- | --- |
| **Baseline** |  |  |  |  |
| Singing session group |  |  |  |  |
| N | 131 | 132 | 132 | 102 |
| Mean (SD) | £21.60 (£44.78) | £21.56 (£47.49) | £26.26 (£56.04) | 0.749 (0.165) |
| Control group |  |  |  |  |
| Number of mothers |  | 59 | 60 | 40 |
| Mean (SD) | £19.07 (£21.13) | £14.57 (£37.08) | £27.73 (£39.22) | 0.764 (0.226) |
| **Baseline to 6 weeks** |  |  |  |  |
| Singing session group |  |  |  |  |
| N | 107 | 108 | 107 | 109 |
| Mean (SD) | £7.32 (£16.64) | £17.92 (£36.20) | £8.49 (£25.64) | 0.794 (0.182) |
| Control group |  |  |  |  |
| N | 34 | 34 | 34 | 34 |
| Mean (SD) | £4.75 (£10.93) | £6.39 (£18.67) | £10.52 (£18.46) | 0.730 (0.212) |
| **6 weeks to 10 weeks** |  |  |  |  |
| Singing session group |  |  |  |  |
| N | 91 | 94 | 94 | _ |
| Mean (SD) | £8.90 (£21.88) | £11.51 (£33.68) | £15.21 (£32.91) | _ |
| Control group |  |  |  |  |
| N | 32 | 30 | 32 | _ |
| Mean (SD) | £12.95 (£26.84) | £6.92 (£20.12) | £10.89 (£18.45) | _ |
| **10 weeks to 20 weeks** |  |  |  |  |
| Singing session group |  |  |  |  |
| N | 82 | 81 | 81 | 80 |
| Mean (SD) | £6.27 (£24.14) | £9.15 (£30.08) | £5.40 (£12.49) | 0.851 (0.193) |
| Control group |  |  |  |  |
| N | 25 | 25 | 25 | 24 |
| Mean (SD) | £8.32 (£23.16) | £3.32 (£11.62) | £4.86 (£7.59) | 0.772 (0.152) |
| **20 weeks to 36 weeks** |  |  |  |  |
| Singing session group |  |  |  |  |
| N | 72 | 73 | 73 | 73 |
| Mean (SD) | £4.52 (£15.46) | £5.74 (£23.81) | £7.31 (£14.32) | 0.894 (0.121) |
| Control group |  |  |  |  |
| N | 18 | 17 | 18 | 18 |
| Mean (SD) | £9.78 (£16.01) | £0 (£0) | £5.13 (£11.46) | 0.835 (0.121) |

**Table S5: Contrast of mean total cost of NHS care contacts and EQ5D utilities**

|  | Baseline adjusted difference in mean (95% CI) |
| --- | --- |
| Total cost of NHS health care contacts over 36 weeks (mother and baby) | £331 (-£220 to £926) |
| Maternal EQ5D-3L utility score: |  |
| Week 6 | 0.071 (-0.006 to 0.149) |
| Week 20 | 0.068 (-0.003 to 0.136) |
| Week 36 | 0.052 (-0.030 to 0.129) |

**Table S6: Cost-utility analysis^1^**

|  | Mean difference (95% CI) | |
| --- | --- | --- |
| **Total cost (Intervention cost to payer plus cost of health care contacts)** |  | |
| Upper | £870 (£319 to £1466) | |
| Lower | £456 (-£94 to £1052) | |
| **Maternal QALY gain over 36 weeks** | 0.041 (0.009 to 0.072) | |
|  | Lower | Upper |
| Cost per QALY gained over 36 weeks (ICER) | £11,122 | £21,215 |
|  | Probability (%) singing sessions are cost-effective | |
|  | Lower | Upper |
| £20,000 per QALY gained (NICE lower) | 82% | 45% |
| £30,000 (NICE upper) | 93% | 76% |
| £15,000 | 69% | 23% |
