# Supplementary material for: Clinical effectiveness, implementation effectiveness and cost-effectiveness of a community singing intervention for postnatal depressive symptoms, SHAPER-PND: randomised controlled trial
Source: Br J Psychiatry. 2025 Oct 15;227(6):836–45. doi: 10.1192/bjp.2025.10377 (PMC12628126; doi:10.1192/bjp.2025.10377)
Supplement: Bind et al. supplementary material 2 — Bind et al. supplementary material [file S0007125025103772sup002.docx]

**SUPPLEMENTARY METHODS**

Protocol changes since registration

We originally included meeting the criteria for a clinical diagnosis of MDD in the postnatal period (according to the DSM-IV SCID criteria) as an inclusion criterium; however, this greatly limited our recruitment abilities and we subsequently removed it as an official protocol deviation.

Sample size determination

Based on the initial RCT on which M4M is based (1), in order to achieve a power of 80% to detect the same effect-size difference between groups following a 2:1 randomisation process, 232 participants were required for the intervention group and 116 for the control group (total=348). Due to the COVID-19 pandemic and multiple lockdowns which delayed and complicated the recruitment process, our final achieved sample size was lower than anticipated (total=199).

Breathe M4M intervention description

Breathe M4M is a 10-week community singing intervention for mothers with symptoms of PND. The programme was delivered to groups of 8–12 mothers in weekly sessions lasting 1 hour. Mothers began a block of 10-week classes in a cohort and continued with the same group and leader for the duration of the cycle. Classes were free to attend and took place in Children’s Centres or other community venues across South London. Mothers attended with their babies and, due to Covid-19 restrictions, were invited to sit in a socially distanced circle on the floor surrounded by soft play cushions and mats. Mothers and artists were required to adhere to the ongoing Covid-19 guidelines until restrictions were lifted. Classes began with welcome songs and icebreakers, introducing the babies and mothers to one another, and then involved the teaching of a range of songs in from across the world in a range of different languages, encouraging engagement in rounds, multiple parts and harmonies across the 10-weeks. Some songs were accompanied by maracas, drums, hand chimes and other simple instruments that the mothers and babies could play together. Instruments such as guitar and ukulele were also used for a small number of songs. Mothers were invited to access Mothers were also invited to join a WhatsApp group so they could keep in contact between sessions. Classes were led by specialist creative health music leads trained by Breathe, with support from a Breathe staff member trained in safeguarding and with experience of working with vulnerable people.

Demographics

We collected, via the baseline online call, the following questionnaires in interview format: baseline demographics on sociodemographic and economic circumstances, maternal and infant health history, obstetric and delivery information, and participation in any other mother-baby activities; the Threatening Life Experiences Questionnaire (2), a validated questionnaire that ascertains experience of stressful life events in the perinatal period; the Child Experience of Care and Abuse Questionnaire (3), a validated interview that assesses for childhood experience of physical and sexual abuse, antipathy, and neglect; the Composite Abuse Scale-Pregnancy Version (4), a validated questionnaire that ascertains experience of intimate partner violence; and, the Intrusive Life Events Scale (5), a validated questionnaire that asks about distressing life events at any point. Participants were then re-administered demographic questionnaires at weeks 6, 10, 20, and 36 via online questionnaires.

Additional measures

*Additional clinical measures*

We collected the Hamilton Depression Rating Scale (6) in interview format at baseline and week 10. Additionally, in order to evaluate further aspects of mental health, wellbeing, and social support, the following scales were collected via online questionnaires at baseline, weeks 6, 10, 20, and 36: the Beck Depression Inventory (7) , the Office for National Statistics Wellbeing Scale (8), the State-Trait Anxiety Inventory (9), the Perceived Stress Scale (10), the UCLA Loneliness Scale (11), the Multidimensional Scale of Perceived Social Support (12), and the Short General Self-Efficacy Scale (13) .

*Biological samples*

In order to assess biological changes throughout the course of the study, mothers and their babies provided saliva samples and mothers provided hair samples. At baseline and week 10, mothers and their babies took saliva samples at home to assess diurnal patterns of cortisol.  Mothers took six saliva samples from themselves using absorbent swabs at awakening, +15, +30, +60, noon, and 8 pm, and took two samples from their babies using passive drool method at awakening and 8pm. At baseline and week 10, mothers and their babies also provided saliva samples at the start and end of their session to assess for cortisol, inflammatory markers, and oxytocin. Furthermore, at week 10, mothers provided hair samples to assess for cortisol output over the previous three months.

*Mother-infant videos*

To evaluate changes in the mother-infant relationship, we collected, via the baseline, week 10, and week 36 online call, mother-infant interaction videos. Mothers were instructed to interact with their babies as they normally would and were recorded for three minutes while the researchers turned their cameras off so as to minimise their presence. Videos were coded for the quality of the mother-infant relationship using the Crittenden Care Index (14) and for maternal mentalisation using the Parental Cognitive Attributions Mentalisation Scale.

Implementation effectiveness

Quantitative data on the implementation effectiveness of both the M4M singing intervention as well as the control activities were collected using: the perceived Acceptability of Intervention Measure (AIM), which assesses whether participants view the activity to be agreeable, palatable, and satisfactory; the perceived Intervention Appropriateness Measure (IAM), which assesses whether participants view the activity to be fit, relevant, and suitable for the management of PND; and the perceived Feasibility of Intervention Measure (FIM), which assesses whether participants view the activity to be practically doable and useable (15). Each measure contains four questions rated on a Likert-scale from 1 to 5, with higher scores indicating higher acceptability, appropriateness and feasibility. All three measures were administered to mothers mid-intervention (week 6) via online questionnaires. Qualitative interviews were also conducted in a sub-sample of mothers to explore acceptability, appropriateness, and feasibility of the singing intervention, results of which are reported in a separate paper (16).

Statistical analyses

Descriptive analyses were conducted with SPSS Statistics version 27 for MacOS (IBM, UK), while linear mixed effects models with multiple imputation were conducted with R version 4.4.2 using the lme4 (v1.1.35.5), and emmeans (v1.10.6) packages. Before analysis, data were checked for normality. Demographics were analysed using frequencies and descriptives.

As described in the protocol, our primary objective was to test whether singing reduces the severity of symptoms of PND on the EPDS between baseline and week 10 (end of intervention) by paired test, followed by a comparison of this outcome between the two groups.

We estimated the treatment effect of singing group relative to control on the change in EPDS scores from eligibility assessment to weeks 6, 10, 20, and 36, adjusting for important determinants of recovery at baseline identified a-priori: 1) history of childhood abuse (CECA), and 2) major depressive disorder diagnosis (antenatal and postnatal). Collectively we refer to these as “baseline covariates”. We included all post-randomisation EPDS scores as the model outcome, and tested treatment effects on change through inclusion of eligibility EPDS as a linear covariate. Linear mixed effects models were used to account for repeated measures. The model was specified: *EPDS_post_ ~ EPDS_eligibility_ + BaselineCovariates + TimePoint + TreatmentGroup + TimePoint×TreatmentGroup + (1 | participant-id),* with subscript “post” indicating measurement at the four post-treatment onset timepoints (weeks: 6, 10, 20, 36). Timepoint was treated as an unordered factor.

Missing outcome data was dealt with using two approaches: 1) intention to treat (ITT; using last observation carried forward) and 2) all available data i.e. applying the same linear mixed effects model to all observed post-treatment data. For the ITT analysis we additionally used multiple imputation (mice package, v3.17.0; 50 imputations with the random forest method) to impute missing values of baseline covariates: CECA (n=7, 3.5%), depression history (n=23, 11.6%). Eligibility EPDS scores were completely observed.

Overall treatment effects, and interactions with time, were tested by model comparison against reduced models removing first the TimePoint×TreatmentGroup interaction, and then the main effect of TreatmentGroup. Treatment effects at individual timepoints were tested using estimated marginal means. For multiply imputed models statistics were pooled using Rubin’s rules, with the D1 method for model comparison. Means and standard deviations, as well as frequencies and percentages, are reported in Table 1. Means and standard error of the mean of EPDS scores are presented in the Results section. EPDS score at weeks 6, 10, 20, and 36 presented relative to each randomisation group’s mean score at eligibility is presented in Figure 2. Treatment effects of singing relative to the control group at weeks 6, 10, 20, and 36 are presented in Figure 3. Implementation effectiveness measures were summed and averaged for each measure, with medians and min-max ranges reported. As data was non-parametric, group medians were compared using a Mann-Whitney U Test.

Cost-effectiveness methods

*Study design*

This was an economic evaluation of the Melodies for Mums program. It combined data on the provider cost of implementing and delivering singing sessions with evidence from the M4M clinical trial on the effect of random assignment to singing sessions on maternal health-related quality- of- life and the cost of health care utilisation for mothers and their babies.

The economic analysis was guided by the National Institute for Health and Care Excellence (NICE) reference case for undertaking economic assessment of health care programmes (17) . We used incremental cost-utility analysis (CUA) to evaluate programme cost-effectiveness, comparing the total cost impact with the benefit of randomisation to singing sessions, quantified as quality-adjusted life year (QALY) gains. Cost and QALY outcomes were then combined to deliver an estimate of the incremental cost per QALY gained associated with the M4M programme (the programme incremental cost-effectiveness ratio – or “ICER”). The ICER was then compared to a range of cost-effectiveness threshold values, including those currently used by NICE when forming guidance on whether new health programmes should be funded by the NHS (18) . We also compared the programme ICER to a threshold argued to be closer to the true opportunity cost of additional NHS expenditure on new health programmes (19).

Costs were quantified from an NHS and local authority (LA) public health payer perspective, assuming that all provider costs would be wholly compensated through either NHS or LA funding streams. The comparator for the economic evaluation was usual practice in the absence of singing sessions and corresponds to the control arm built into the statistical design of the M4M clinical trial.

The time horizon for the CUA was limited to a 36-week period corresponding to the trial follow-up period. Reported cost and QALY outcomes are undiscounted given that the trial study period for each participant was less than 12-months.

*Provider costs*

The M4M provider supplied estimates of costs that would be incurred in providing a program consisting of 20 singing sessions over a 10-week period, reaching 60 individual mothers and babies enrolled to participate (or 30 mother-baby dyads).  Costs relate to a range of program activities: initial population scoping work in areas where singing sessions are not currently offered; programme planning, training of artists, organisational overheads, and resource inputs needed to deliver classes, including artist fees. This information was used to estimate a cost per enrolled mother/baby dyad used and used to undertake the CUA. Two per dyad provider costs were estimated: a “base case cost” that includes the cost of all activities and expenditure arising from programme implementation and delivery; and an alternative estimate based only costs relating to the direct delivery of singing sessions and provider overheads. The alternative value is intended to facilitate like-for-like comparisons with published trial-based ICERs for other programmes which would typically not include wider programme costs (20), including activities relating to implementation (e.g. scoping work, programme development etc.).

*Wider NHS care utilisation*

Participant level data from a Demographic Questionnaire administered to mothers participating in the clinical trial was used to measure the quantity of maternal and infant health care utilisation over a 36-week period.  This included questions about frequency of contact with community- and hospital-based NHS services pertaining to maternal and infant health (including GP visits, Accident and Emergency (A and E) visits, outpatient attendance and inpatient admissions). Mothers were also asked to report how many times they had attended NHS psychological therapy. The questionnaire was administered either face-to-face or by telephone across 5 time points: baseline, 6, 10, 20 and 36-weeks post-randomisation.

*Unit costs*

Costs of contacts with NHS care professionals and services were obtained from published sources (<https://www.england.nhs.uk/costing-in-the-nhs/national-cost-collection/#ncc1819>; PSSRU). A list of all unit costs used in the analysis are reported in the Supplementary Tables.

*Calculation of care utilisation costs*

The cost of NHS care utilisation over each trial period was estimated by multiplying the number of self-reported care contacts of a specific type for mother or baby by its corresponding unit cost. Cost of care contacts were initially standardised to weekly values to allow for differences in the length of measurement periods within the clinical trial.

All costs (provider programme costs and cost of care utilisation) are presented in pounds sterling and reported at 2023/2024 price levels.

*Maternal QALYs*

Maternal health-related quality of life was measured using the EQ-5D-3L instrument (21) administered at baseline, 6, 20, and 36 weeks (no data was collected at 10 weeks). The instrument was administered either face-to-face or via on-line self-completion. Participants report across five health-related quality-of-life domains (mobility, self-care, usual activities, pain, and anxiety/depression), rating current impairment experienced (‘none’ to ‘extreme’). Based on these responses, each participant is assigned to one of 243 possible quality of life states. ‘Utility’ scores applicable to each state were then used to estimate a quality-adjusted life year (QALY) value for each participant over a 36-week period. The utilities are based on an existing UK survey of public preferences for different quality of life states (22), with scores ranging from a maximum value of 1 (corresponding to “full health”) to 0 (death) with negative values describing states considered worse than being dead.

*Statistical modelling of per dyad care utilisation costs and maternal utilities*

Statistical analysis of economic data from the clinical trial was carried out on an intention (ITT) to treat basis. Random intercept linear mixed models (LMM) (23,24) were used to contrast differences between trial arms in average (mean) weekly cost of care contacts for mother and baby separately and the weekly total cost of all care contacts per dyad over each trial period (the latter outcome used within the main CUA). LMM was also used to compare mean maternal EQ5D utility scores reported at the end of each trial period. All models included a categorical variable indicating the trial time point and an interaction between time and a binary covariate identifying trial allocation (“intervention” or “control”). The coefficient on this interaction was used to identify the cost and utility scores contrasts between groups. Additional baseline covariate adjustments included: a baseline predictor of missing outcome data (participant ethnicity) and trial stratification variables (EDS scores and age of baby). LMM group comparisons are valid under the assumption that missing outcome data at each measurement point are missing at random (MAR), conditional on observable outcomes and selected baseline covariates.

*Combing cost and outcome data*

The LMMs were bootstrapped 5000 times to generate a joint distribution of mean weekly total cost of NHS care utilisation and utility scores predicted for both trial groups for each trial period. At this post-estimation stage a distribution of mean QALY values over 36-weeks was arrived at by applying the area under the curve method (25) to the distribution of predicted mean utility scores. Predicted weekly care costs were summed to determine the cost per trial period and then then added together across periods to derive a distribution of costs covering the entire 36-week trial period. For the singing sessions group, the provider programme cost was added to these total care utilisation costs. A joint distribution of the group difference in mean total cost and QALYs was then determined and used to identify the programme ICER based on the respective mean values of the differences.

𝐼𝐶𝐸𝑅=(𝑀𝑒𝑎𝑛 𝑡𝑜𝑡𝑎𝑙 𝑐𝑜𝑠𝑡𝑠𝑖𝑛𝑔𝑖𝑛𝑔)−(𝑀𝑒𝑎𝑛 𝑡𝑜𝑡𝑎𝑙 𝑐𝑜𝑠𝑡𝑢𝑠𝑢𝑎𝑙 𝑐𝑎𝑟𝑒)(𝑀𝑒𝑎𝑛 𝑄𝐴𝐿𝑌𝑠𝑠𝑖𝑛𝑔𝑖𝑛𝑔)−(𝑀𝑒𝑎𝑛 𝑄𝐴𝐿𝑌𝑠𝑢𝑠𝑢𝑎𝑙 𝑐𝑎𝑟𝑒)ICER=(Mean total costsinging)−(Mean total costusual care)(Mean QALYssinging)−(Mean QALYsusual care)

We present two ICERs: a base case and alternative estimate corresponding to our base case and alternative programme provider cost estimates.

Uncertainty due to trial sampling error was characterised using probabilities. We present the probability of total cost and QALYs over 36-weeks being higher for the singing session group based on the proportion of the distribution of cost and outcome differences with positive values. The probability that singing sessions were cost-effective over 36-weeks was inferred from the proportion of the joint distribution of cost and QALY outcomes implying a cost-effective outcome at different cost-effectiveness threshold values.

*Sensitivity analysis*

We evaluated the sensitivity of the base case and alternative ICER to a reduction in assumed population reach (numbers of mothers recruited to participate in sessions) as this will impact directly on the estimated programme cost per mother/baby dyad. Specifically, we reduce the assumed level of reach by a third, from 30 mother/baby dyads to 20 recruited for 20 sessions over 10 weeks.   All analyses were performed in Stata (version 17). Reporting follows CHEERS guidelines (26).

The total incremental cost (NHS intervention plus wider care contact costs) per maternal QALY gained ranged from £11,122 to £21,215, depending on the whether the lower or the upper intervention payer cost was applied. This range almost entirely falls below what the UK health care regulator (National Institute for Health and Care Excellence, NICE) recommends the NHS should pay for health care programmes (a maximum of £20,000 to £30,000 per QALY gain) (27) . In sensitivity analysis, a modest (one third) reduction in assumed numbers of mother-baby pairs recruited to singing sessions (increasing the intervention cost per dyad) had little effect on the lower cost-per QALY estimate (increasing to £12,659) and a more substantive effect on the upper estimate (up to £27,780). Our findings also carry some margin for error due to trial sampling error. This increased with the level intervention cost assumed to borne by the NHS/public health payer. At the upper payment value, the probability that the intervention was cost-effective using the NICE criterion ranged from 45% to 73%, depending on whether the bottom (£20,000) or the top (£30,000) NICE threshold value is applied. At the lower payment value uncertainty diminishes, with corresponding probabilities of 82% and 93%.

**SUPPLEMENTARY DISCUSSION**

The economic evaluation had limitations: missing data were extensive, and consequently we cannot rule out the possibility that this may introduce bias to estimates. Moreover, we also could not evaluate all relevant economic outcomes over a longer time-frame, such as prevention of admission to expensive PND care facilities due to stabilisation of symptoms, or any positive impact on infant and child health and development, so the reported values may be conservative based on the increasing difference between the groups over the weeks following the end of the intervention. Comparison with other published cost-effectiveness studies of PND interventions should be made cautiously because of differing health systems and perspectives, scope of measurement, comparators and time-horizons. Finally, while mothers in the singing group may have utilised more healthcare services during the study, we were unable to establish whether these initial increased costs ultimately saved the NHS money in the long-term.

**REFERENCES**

1. Fancourt D, Perkins R. Effect of singing interventions on symptoms of postnatal depression: three-arm randomised controlled trial. The British Journal of Psychiatry. 2018;212(2):119–21.

2. Brugha TS, Cragg D. The list of threatening experiences: the reliability and validity of a brief life events questionnaire. Acta Psychiatr Scand. 1990;82(1):77–81.

3. Bifulco A, Bernazzani O, Moran PM, Jacobs C. The childhood experience of care and abuse questionnaire (CECA.Q): Validation in a community series. British Journal of Clinical Psychology. 2005 Nov;44(4):563–81.

4. Hegarty K, Sheehan M, Schonfeld C. A multidimensional definition of partner abuse: development and preliminary validation of the Composite Abuse Scale. In: Domestic Violence. Routledge; 2017. p. 15–31.

5. Bebbington PE, Bhugra D, Brugha T, Singleton N, Farrell M, Jenkins R, et al. Psychosis, victimisation and childhood disadvantage: evidence from the second British National Survey of Psychiatric Morbidity. The British Journal of Psychiatry. 2004;185(3):220–6.

6. Williams JBW. A structured interview guide for the Hamilton Depression Rating Scale. Arch Gen Psychiatry. 1988;45(8):742–7.

7. Beck AT, Ward C, Mendelson M, Mock J, Erbaugh J. Beck depression inventory (BDI). Arch Gen Psychiatry. 1961;4(6):561–71.

8. Benson T, Sladen J, Liles A, Potts HWW. Personal Wellbeing Score (PWS)—a short version of ONS4: development and validation in social prescribing. BMJ Open Qual [Internet]. 2019 Apr 1;8(2):e000394. Available from: <http://bmjopenquality.bmj.com/content/8/2/e000394.abstract>

9. Spielberger CD. Manual for the State-Trait Anxiety Inventory STAI (form Y)(“ self-evaluation questionnaire”). 1983;

10. Cohen S, Kamarck T, Mermelstein R. Perceived stress scale. Measuring stress: A guide for health and social scientists. 1994;10:1–2.

11. Russell DW. UCLA Loneliness Scale (Version 3): Reliability, validity, and factor structure. J Pers Assess. 1996;66(1):20–40.

12. Wilcox S. Multidimensional Scale of Perceived Social Support. Psychol Trauma. 2010;2(3):175–82.

13. Romppel M, Herrmann-Lingen C, Wachter R, Edelmann F, Düngen HD, Pieske B, et al. A short form of the General Self-Efficacy Scale (GSE-6): Development, psychometric properties and validity in an intercultural non-clinical sample and a sample of patients at risk for heart failure. GMS Psycho-Social-Medicine. 2013;10.

14. Crittenden PM. CARE-Index manual. Unpublished manuscript, Miami: Family Relations Institute. 2003;

15. Weiner BJ, Lewis CC, Stanick C, Powell BJ, Dorsey CN, Clary AS, et al. Psychometric assessment of three newly developed implementation outcome measures. Implementation Science [Internet]. 2017 Feb 5;12(1):108. Available from: <https://doi.org/10.1186/s13012-017-0635-3>

16. Han E, Davis R, Soukup T, Bradbury A, Williams J, Lopez MB, et al. Implementation of singing groups for postnatal depression: experiences of participants and professional stakeholders in the SHAPER-PND randomised controlled trial. 2025 Feb 18 [cited 2025 Feb 25]; Available from: <https://osf.io/tp8g9_v1>

17. Guide to the Methods of Technology Appraisal 2013 [Internet] - PubMed [Internet]. [cited 2025 Feb 18]. Available from: <https://pubmed.ncbi.nlm.nih.gov/27905712/>

18. The Guidelines Manual [Internet] - PubMed [Internet]. [cited 2025 Feb 18]. Available from: <https://pubmed.ncbi.nlm.nih.gov/27905714/>

19. Claxton K, Martin S, Soares M, Rice N, Spackman E, Hinde S, et al. Methods for the estimation of the National Institute for Health and Care Excellence cost-effectiveness threshold. Health Technol Assess [Internet]. 2015 Feb 1 [cited 2025 Feb 18];19(14):1. Available from: <https://pmc.ncbi.nlm.nih.gov/articles/PMC4781395/>

20. Johns B, Baltussen R, Hutubessy R. Programme costs in the economic evaluation of health interventions. Cost Effectiveness and Resource Allocation [Internet]. 2003 Feb 26 [cited 2025 Feb 18];1(1):1–10. Available from: <https://link.springer.com/articles/10.1186/1478-7547-1-1>

21. Group TE. EuroQol-a new facility for the measurement of health-related quality of life. Health Policy (New York). 1990;16(3):199–208.

22. Dolan P. Modeling valuations for EuroQol health states. Med Care. 1997;35(11):1095–108.

23. Faria R, Gomes M, Epstein D, White IR. A Guide to Handling Missing Data in Cost-Effectiveness Analysis Conducted Within Randomised Controlled Trials. Pharmacoeconomics [Internet]. 2014 Nov 26 [cited 2025 Feb 18];32(12):1157–70. Available from: <https://link.springer.com/article/10.1007/s40273-014-0193-3>

24. Gabrio A, Plumpton C, Banerjee S, Leurent B. Linear mixed models to handle missing at random data in trial-based economic evaluations. Health Econ [Internet]. 2022 Jun 1 [cited 2025 Feb 18];31(6):1276–87. Available from: <https://onlinelibrary.wiley.com/doi/full/10.1002/hec.4510>

25. Economic Evaluation in Clinical Trials - Henry A. Glick, Jalpa A. Doshi, Seema S. Sonnad, Daniel Polsky - Google Books [Internet]. [cited 2025 Feb 18]. Available from: <https://books.google.co.uk/books?hl=en&lr=&id=Xqi1BAAAQBAJ&oi=fnd&pg=PP1&dq=Glick+HA,+Doshi+JA,+Sonnad+SS,+Polsky+D.+Economic+Evaluation+in+Clinical+Trials.+Oxford+University+&ots=fp7d1vcdHS&sig=GWR7D-YH3vUGap2Pw4nHeP-ODSI&redir_esc=y#v=onepage&q&f=false>26. Husereau D, Drummond M, Petrou S, Carswell C, Moher D, Greenberg D, et al. Consolidated Health Economic Evaluation Reporting Standards (CHEERS)—Explanation and Elaboration: A Report of the ISPOR Health Economic Evaluation Publication Guidelines Good Reporting Practices Task Force. Value in Health. 2013 Mar 1;16(2):231–50.

27. McCabe C, Claxton K, Culyer AJ. The NICE cost-effectiveness threshold: what it is and what that means. Pharmacoeconomics. 2008;26:733–44.
